# Evaluation of the *Conversations About Gambling* Mental Health First Aid course: effects on knowledge, stigmatising attitudes, confidence and helping behaviour

**DOI:** 10.1186/s40359-022-00785-w

**Published:** 2022-03-24

**Authors:** Kathy S. Bond, Fairlie A. Cottrill, Amy J. Morgan, Kathryn J. Chalmers, Julia N. Lyons, Alyssia Rossetto, Claire M. Kelly, Louise Kelly, Nicola J. Reavley, Anthony F. Jorm

**Affiliations:** 1 Mental Health First Aid Australia, 369 Royal Parade, Parkville, VIC 3052 Australia; 2 Centre for Mental Health, Melbourne School of Population and Global Health, The University of Melbourne, Carlton, VIC 3010 Australia

**Keywords:** Problem gambling, Mental health first aid, Public intervention, Education, Early intervention

## Abstract

**Background:**

The effects of problem gambling are wide-ranging, affecting many aspects of health and negatively impacting the person who gambles, their family and friends, and their community. People experiencing problem gambling have low rates of help-seeking and perceive many barriers to treatment, although evidence suggests that encouragement and support from friends and family can increase rates of help-seeking. Mental Health First Aid Australia’s *Conversations About Gambling* course aims to teach members of the public evidence-based strategies for recognising and responding to signs of problem gambling in a person they know.

**Methods:**

This research evaluated the effects of the *Conversations About Gambling* course on participants’ knowledge, confidence, stigmatising attitudes, intended helping behaviour and actual helping behaviour towards a person experiencing problem gambling. Participants from Australia completed surveys before the course, immediately after the course and six months later. Changes over time (pre-course to post-course, and pre-course to 6-month follow-up) were assessed with linear mixed models. Descriptive statistics and content analyses of open-ended questions pertaining to participants’ satisfaction with the course were also produced.

**Results:**

Between 2018 and 2020, 166 participants were recruited into this study. At 6-month follow-up 87 participants (52.4%) provided data. Participants’ knowledge about gambling and gambling problems, confidence, desire for social distance and intentions to help a person experiencing problem gambling significantly improved from pre-course to post-course, and from pre-course to 6-month follow-up. The quality of some actions taken to support a person they knew who was experiencing problem gambling also improved from pre-course to 6-month follow-up, in line with the teachings of the course. Participants perceived the course to be highly acceptable.

**Conclusions:**

The results of this initial evaluation of Mental Health First Aid Australia’s *Conversations About Gambling* course suggest that it is an effective and acceptable educational intervention for those who wish to support a person experiencing problem gambling.

**Supplementary Information:**

The online version contains supplementary material available at 10.1186/s40359-022-00785-w.

## Introduction

Gambling activities are a form of entertainment where a person is required to “risk losing something of value for the chance of winning more” [1]. Gambling can become addictive and lead to problem gambling, defined as difficulties in limiting money and/or time spent on gambling, with subsequent negative impacts on the gambler, their family and friends, or the community [2]. The term “problem gambling” encompasses both a diagnosable disorder and behaviours or symptoms which have a substantial adverse effect on the gambler’s life despite not meeting the threshold for formal diagnosis. While the international literature uses various terms and definitions to describe this phenomenon, in this paper the term “problem gambling” will be used, in line with Australia’s national definition [2] and research outputs.

While more than 60% of Australian adults have stated they partake in gambling, an estimated 0.4% of adults are considered to have problem gambling, with higher percentages of adults at low (3.0%) or moderate risk (1.9%) of problem gambling [3]. People with problem gambling have reported experiencing financial and legal difficulties, disruptions to work and/or study, psychological distress, and relationship conflict or breakdown [4]. They are also at higher risk of developing other mental health problems compared to the general population. An international systematic review found that there were higher rates of substance use disorders (58%), mood disorders (38%) and anxiety disorders (37%) amongst people experiencing problem gambling [5]. Their family and friends report experiencing relationship distress, financial and legal difficulty, neglect of children, and intimate partner and family violence [4]. Suicidal thoughts and behaviours are also more prevalent in people with problem gambling and their family members [6]. Problem gambling can also affect the community through cultural harms (by limiting participation in and engagement with cultural roles, practices and communities), and increases in criminal activity [4].

Despite the diverse and numerous consequences of problem gambling, behaviours consistent with problem gambling can often be concealed from family or friends, meaning that many people are unaware of the magnitude of a person’s problem gambling, or that they are gambling at all [7]. This makes it difficult for family and friends to encourage the person to seek help. One resource that has been developed to identify the signs of problem gambling in the gambling venue is the Gambling Behaviour Checklist [8–10]. This is a validated checklist used by gambling venue staff to identify whether patrons are displaying risky or problematic gambling behaviours [8–10]. These include losing control over gambling, seeking funds to gamble, gambling intensely, gambling for a long duration, displaying superstitious behaviour, having an emotional response to losing, and displaying unusual social behaviour (e.g. avoidance of contact or conversation with other people). An evaluation of the Gambling Behaviour Checklist with gambling venue staff who have limited work experience showed that it increased their confidence in identifying and approaching gamblers and the frequency of follow-up action, typically in the form of an informal chat with the gambler [8].

People experiencing problem gambling typically have low rates of seeking professional help and professional help-seeking is typically preceded by seeking informal help or self-help [11]. They perceive several barriers that prevent them from accessing effective treatment, including an unwillingness to admit to the problem [12], feelings such as shame and embarrassment [12], minimising the extent of their gambling problems [12], stigma towards mental illnesses and problem gambling [13], concerns about treatment quality and/or effectiveness, and a lack of clarity around treatment options [12]. Research has found that rates of professional help seeking increase with an increase in the number of symptoms of problem gambling [14]. Motivators for seeking help often revolve around the degree of harm caused to people in the person’s life. The majority of people experiencing problem gambling have sought help after experiencing a crisis such as a financial or family crisis [15]. Declining physical and psychological health is also a factor in seeking effective treatment [15]. Encouragement from family and friends also improves low rates of help seeking behaviour, however, few receive this encouragement [16]. Recommendations made by Evans and Delfabbro [15] and Hing, Nuske and Gainsbury [11] suggest that community training—involving family, friends and co-workers—should be utilised to recognise the signs of problem gambling and how to support, give advice, and encourage professional help seeking in a person with problem gambling.

Mental Health First Aid (MHFA) is an early intervention program that uses evidence-based guidelines to inform the curriculum of its training courses [17]. MHFA training, developed by MHFA Australia, educates members of the public about mental illnesses such as depression, anxiety, psychosis and substance abuse, and mental health crises such as suicidal thoughts and behaviours and non-suicidal self-injury. It is also tailored to settings (e.g. workplace and tertiary students) and to stages of life, and is culturally sensitive, with specific courses for Aboriginal and Torres Strait Islander people. Evaluations of the Standard and Youth face-to-face MHFA courses show that participants report improved confidence in helping someone experiencing a mental health crisis, greater knowledge regarding mental health, fewer negative attitudes, and demonstrate greater supportive behaviours toward people with mental health problems [18, 19].

Given that the Standard MHFA course does not cover problem gambling, and there is limited scope and time to adequately address this topic within the existing structure of the course, MHFA Australia have developed a new course that specifically addresses problem gambling and associated first aid skills. Using the Delphi expert consensus method, Bond and colleagues [20] developed guidelines on how friends and family can help a loved one with problem gambling. A total of 66 people (34 with lived experience and 32 professionals) from Australia, North America, New Zealand and the United Kingdom participated in the Delphi study. Results yielded 234 endorsed statements from a total of 412 generated from a literature search. These statements informed the development of MHFA Australia’s *Conversations About Gambling* course. This course, formerly named *MHFA for Gambling Problems*, is a 4-h face-to-face course for the general public that teaches practical skills to support someone with problem gambling, including how to encourage professional help seeking.

Evaluating the outcomes of any course developed by MHFA Australia is an important process to ensure participants acquire and retain knowledge, attitudes and skills that are consistent with the recommendations made in the guidelines [20]. It also allows adjustments to be made to the course curriculum, if necessary. The aim of this evaluation of the *Conversations About Gambling* course is to assess the participants’ knowledge about gambling and gambling problems, their stigmatising attitudes towards a person experiencing problem gambling, their confidence in helping and quality of helping behaviours, and course satisfaction.

## Methods

### Intervention

*Conversations About Gambling* is a four-hour course that teaches participants the skills and knowledge required to provide appropriate support to a person who is experiencing problem gambling. The course is based on best practice guidelines developed through the expert consensus of international lived experience advocates and gambling help professionals [21]. All community members are eligible to enrol in this course, regardless of whether they have completed other MHFA courses.

The course is facilitated by an Instructor accredited by MHFA Australia. The course materials include PowerPoint slides, videos, interactive group and individual activities, and a handbook [22]. The course learning objectives are:

- To gain an understanding of the prevalence of gambling in Australia
- To gain an understanding about the continuum of gambling and risk factors associated with problem gambling
- To be able to identify signs that indicate someone may be experiencing problem gambling
- To understand motivations for gambling and the relationship between problem gambling and mental health problems
- To be able to apply mental health first aid skills to a person experiencing problem gambling
- To learn crisis first aid for suicidal thoughts and behaviours
- To understand relapse in the context of problem gambling.

### Procedures

Participants were recruited from *Conversations About Gambling* courses that were conducted in Australia between 2018 and 2020. A research officer contacted MHFA Instructors for permission to collect evaluation data from courses they were delivering in Australian capital cities. At the beginning of these courses, a research officer invited participants to enrol in the study and complete paper surveys before and immediately after the course. Participants were also contacted via email one week prior to the course and offered the opportunity to complete the pre-course survey online (n = 17). Participants who agreed to participate in the study were emailed a link to the follow-up survey six months after the course. All online surveys were hosted by SurveyMonkey. Participants who had not completed the follow-up survey received three email reminders and one phone call reminder.

## Measures

### Demographic information

The pre-course survey gathered demographic information, information about previous training in mental health and gambling problems, and personal and professional experience of gambling problems.

### Knowledge about gambling and gambling problems

At each measurement occasion, participants were presented with 20 true or false statements about gambling and gambling problems, derived from the course content, and were asked to respond “Disagree”, “Agree” or “Don’t know”. A “Don’t know” response was marked as an incorrect response. Knowledge scores were calculated based on the percentage of correct answers and could therefore vary between 0 and 100. The coefficient alpha was 0.78.

### Confidence in supporting a person experiencing problem gambling

On each occasion of measurement, participants were presented with a vignette about a woman named Patricia who is experiencing gambling problems (see Additional File 1 for copies of the vignette and each survey). The vignette was developed using DSM-5 criteria for a gambling disorder. The same vignette was used at each time point to enable a reliable assessment of change over time, and to minimise potential confounders (e.g., discrepancies in the difficulty or interpretation of different vignettes). Participants were asked how confident they were in their ability to help Patricia on a 5-point Likert scale from “Not at all confident” (a score of 1) to “Extremely confident” (a score of 5).

### Social distance

At each measurement occasion, participants were asked to what extent they agreed with seven statements designed to measure social distancing attitudes (i.e. people’s willingness to interact with or avoid) towards “people like Patricia”. Responses were reported on a 5-point Likert scale ranging from “Definitely not” (a score of 1) to “Yes, definitely” (a score of 5). This measure is based on the Social Distance Scale by Link and colleagues [23] and had a Cronbach’s alpha of 0.86.

### Quality of intended helping behaviours

Intended help for a person with problem gambling was measured by asking about the likelihood that participants would take each of 19 actions in assisting a person like Patricia. Responses were provided on a 5-point Likert scale (“Very unlikely” to “Very likely”). Eleven actions were consistent with the evidence and the teachings of the course, while eight were not recommended. Separate scales for recommended and non-recommended actions were created. Concordance with the training was assessed by calculating the number of recommended actions participants rated themselves as “Likely” or “Very likely” to do (range 0 to 11). Conversely, for actions that were not recommended, concordance with training was calculated by the number of actions participants rated themselves as “Unlikely” or “Very unlikely” to do (range 0 to 8). A cut-off score of 80% of actions that were concordant with training was set to indicate mastery of intended support. For recommended actions, mastery was indicated by scores of 9 and above. For non-recommended actions, mastery was indicated by a score of at least 7 (i.e. participants did not intend to do at least 7 of the non-recommended actions). As these are criterion-referenced tests, we calculated the agreement coefficient as a measure of reliability. We used Subkoviak [24] to estimate the agreement coefficient based on a single administration of the measure. The agreement coefficient was 0.76 for recommended actions and 0.71 for non-recommended actions. There was no significant correlation between recommended and non-recommended intended actions (r = 0.07, 95 CI: −0.09 to 0.23).

### Confidence in and quality of actual helping behaviours

At pre-course and 6-month follow-up, participants were asked whether they had known anyone showing signs of problem gambling in the past 6 months and what they had done to help the person they had had the most contact with. Participants were asked how confident they had been in their ability to help the person using a 5-point Likert scale.

Participants were also asked whether they had done any of 20 actions to help the person showing signs of problem gambling that they had had the most contact with. These actions mirrored the intended help actions described above, with one additional item (“I did not do anything”). Eleven actions were consistent with the evidence and the teachings of the course, while nine (including “I did not do anything”) were not recommended. Separate scales for recommended and non-recommended actions were created. Concordance with the training was assessed by calculating the number of recommended actions the participant reported doing (range 0 to 11) and the number of non-recommended actions they avoided doing (range 0 to 9). A cut-off score of 80% of actions that were concordant with training was set to indicate mastery of support. For recommended actions, a score of at least 9 indicated mastery of the scale. For non-recommended actions, mastery was indicated by a score of at least 8 (i.e. participants avoided doing at least 8 of the non-recommended actions). The agreement coefficient was 0.93 for recommended actions and 0.86 for non-recommended actions. There was no significant correlation between recommended and non-recommended actions (r = -0.07, 95 CI: −0.26 to 0.12).

### Course satisfaction

In the post-course survey, participants were asked to rate their satisfaction with the *Conversations About Gambling* course. Using a 5-point Likert scale, they indicated how new, how understandable and how relevant the information was, how well it was presented and their satisfaction with the course materials. They were also asked open-ended questions about the strengths and weaknesses of the course.

### Statistical analysis method

The data were analysed with linear mixed models. Models included a fixed effect of time, and random effects of participant and course, to adjust for the correlation of responses within participants over time and within courses. Mixed models retain all available data and yield an intention-to-treat estimate of change under the assumption that data are missing at random [25]. Logistic regression models investigated whether missing at follow-up was associated with any demographic variables (gender, age, education, Aboriginal or Torres Strait Islander, language spoken at home, previous training in gambling problems, previous training in mental health) or pre-course outcome variables. As participants with previous training in mental health were significantly less likely to be missing at follow-up, this variable was also included as a fixed effect to help meet the missing at random assumption. Where variables had skewed distributions that yielded skewed residuals and transforming scores was not successful, bootstrapping and calculation of bias-corrected parameter confidence intervals was conducted to assess the robustness of conclusions reached using conventional methods. The main interests were in change over time between pre-course and post-course, and change over time between pre-course and 6-month follow-up. Effect sizes (Cohen’s d) were calculated by dividing the difference between means by their pooled standard deviation, and interpreted according to Cohen’s criteria [26]. Where scales were formed from multiple items, missing responses were imputed as mean values when a respondent had answered at least 80% of the items on the scale. Analyses were performed in Stata 16 and the significance level was set at *p* < 0.05.

## Results

### Participants

Researchers attended 17 courses. Of the 174 course attendees approached, 166 (95.4%) were recruited and 165 provided at least some data immediately after the course. At the 6-month follow-up 87 participants (52.4%) provided data. Participants with previous training in mental health were significantly less likely to be missing at follow-up (39.6% vs 56.2%). No other predictors of missingness were significant.

Table 1 shows participants’ demographic details. The mean age was 41.3 years and nearly three quarters were female. The majority had completed secondary school and had additional formal post-secondary qualifications. Most participants (87.8%) undertook the course for workplace or professional reasons. Nearly a quarter (24.4%) reported having had contact with someone showing signs of problem gambling in the past. A minority nominated supporting someone with problem gambling as the reason for their attendance (16.4%). Over half of the participants (55.5%) had previous mental health training and just over one third (35.6%) had previous training about gambling problems. Many participants also reported having some experience with problem gambling. This was most commonly with clients or customers (41.8%), the broader community (30.3%) and family (27.3%).

**Table 1:** Participant characteristics pre-course (n = 166)

Variable	
Age—M (SD)	41.3 (13.1)
Gender—N (%)	
Male	44 (26.5)
Female	122 (73.5)
Other	0 (0)
Education—N (%)	
Year 9 or lower	3 (1.8)
Year 10, 11, or 12	34 (20.7)
Certificate, Trade or Apprenticeship	52 (31.7)
University	75 (45.7)
Aboriginal or Torres Strait Islander—N (%)	12 (7.4)
Language other than English—N (%)	27 (16.6)
Postcode—N (%)	
Metropolitan area	64 (38.6)
Regional area	100 (60.2)
Missing	2 (1.2)
Previous training about gambling problems—N (%)	58 (35.6)
Previous training in mental health—N (%)	91 (55.5)
Experience with gambling problems—N (%)	
Clients or customers	69 (41.8)
Colleague	16 (9.7)
Myself	4 (2.4)
Friends	37 (22.4)
Family	45 (27.3)
Broader community	50 (30.3)
None of the above	38 (23.0)
Rather not say	1 (0.6)
Reason for learning MHFA for gambling problems—N (%)	
Part of continuing education for workplace/profession	144 (87.8)
Part of training for a volunteer job	13 (7.9)
To support someone with gambling problems	27 (16.4)
Past contact with someone with gambling problems	40 (24.4)
They have had gambling problems	3 (1.8)
Other	9 (5.4)

### Knowledge about gambling and gambling problems

Overall, participants showed a medium level of knowledge about gambling and gambling problems before the course (see Table 2). Knowledge significantly improved immediately after the course and at follow-up, with scores above 80% at both timepoints. These improvements were large in size (see Table 3). Means and standard deviations for knowledge measures are shown in Table 2.

**Table 2:** Observed means and standard deviations for outcome measures

	Pre-course (n = 166)^#^		Post-course (n = 165)^#^		Follow-up (n = 87)^#^	
	Mean	SD	Mean	SD	Mean	SD
Confidence in helping vignette	2.71	0.96	3.95	0.67	3.86	0.79
Social distance	2.73	0.73	2.40	0.80	2.43	0.78
Knowledge about gambling problems	68.11	19.01	85.56	10.28	82.26	12.03
Intended help—recommended actions, number concordant	9.06	2.09	10.19	1.12	9.45	1.90
Intended help—non-recommended actions, number concordant	6.12	1.65	7.06	1.24	6.83	1.36
Help provided—recommended actions, number concordant	4.39	2.97			5.28	2.95
Help provided—non-recommended actions, number concordant	8.51	0.74			8.91	0.34
Confidence in helping person with problem gambling	2.62	1.11			3.62	0.93

^#^Number of observations varies slightly due to missing data

**Table 3:** Mean changes over time from pre-course to post-course, and pre-course to follow-up

	Mean change over time contrasts									
	Pre to post					Pre to follow-up				
	M	95% CI	*p*	d	95% CI	M	95% CI	*p*	d	95% CI
Confidence in helping vignette	1.24	1.10 to 1.39	** < .001**	1.47	1.21 to 1.73	1.12	0.95 to 1.29	** < .001**	1.27	0.99 to 1.56
Social distance	−0.33	−0.42 to −0.24	** < .001**	−0.43	−0.64 to −0.21	−0.25	−0.37 to −0.14	** < .001**	−0.40	−0.66 to −0.13
Knowledge about gambling problems	17.67	15.03 to 20.31	** < .001**	1.14	0.91 to 1.38	13.14	9.83 to 16.46	** < .001**	0.83	0.56 to 1.11
Intended help—recommended actions, number concordant	1.14	0.81 to 1.47	** < .001**	0.68	0.45 to .90	0.42	0.81 to 1.47	**.041**	0.20	−0.07 to 0.46
Intended help – non-recommended actions, number concordant	0.97	0.73 to 1.21	** < .001**	0.64	0.41 to 0.87	0.70	0.41 to 1.00	**.033**^**a**^	0.45	0.19 to 0.72
Help provided—recommended actions, number concordant						0.58	−0.07 to 1.23	.079	0.30	−0.02 to 0.62
Help provided—non-recommended actions, number concordant^b^						0.28	0.14 to 0.47	**.001**	0.63	0.31 to 0.96
Confidence in helping person with problem gambling						0.97	0.71 to 1.24	** < .001**	0.96	0.62 to 1.29

Bolded values indicate a significance level of* p* < .05

a. Value is from model using transformed data to meet model assumptions

b. Bias-corrected parameters based on 2000 bootstrapped replications

### Social distance

Desire for social distance (indicating a willingness to interact with a person with problem gambling) significantly improved post-course (*p* < 0.001) and from pre to follow-up (*p* < 0.001; see Table 3). Improvements were medium in size.

### Quality of intended helping behaviours

#### Recommended actions

Table 4 shows the proportion of participants who intended to do each recommended action at each timepoint. Prior to the course, 69.8% of participants showed mastery of the recommended actions (see Table 4)*.* Percentages generally ranged from 61% (suggesting to leave bank cards and credit cards at home if going to a gambling venue) to over 92% (talking about the behaviours of concern). About half of the actions showed near 100% endorsement immediately after the course, however, this level of endorsement was not sustained at follow-up. The proportion of participants meeting mastery on recommended actions significantly improved from pre-course to post-course (*p* < 0.001), but not from pre-course to follow-up (*p* = 0.170; see Table 3). Furthermore, although there were high rates of concordance with recommended actions before the course (a mean of 9 out of 11 recommended actions), there was a significant improvement both after the course and at follow-up. This improvement was medium-to-large post-course, but only small at follow-up (see Table 3).

**Table 4:** Number and percent of participants who intended to do recommended actions to support Patricia^a^

Recommended actions	Pre		Post		Follow-up	
	n	%	n	%	n	%
5. Give Patricia some information about gambling help services	142	86.1	161	98.2	77	89.5
6. Point out some things that you appreciate about Patricia and your relationship with her	151	91.5	160	98.2	79	91.9
8. Talk with Patricia about the behaviours that are concerning you	153	92.7	156	95.1	80	93.0
12. Suggest she find activities she enjoys that do not involve gambling	131	78.9	150	90.9	62	72.1
13. Involve Patricia in activities that she enjoys that do not involve gambling	153	92.7	163	98.8	80	93.0
14. Encourage Patricia to get support from other people (e.g., family or friends) who are not involved in gambling	145	87.9	160	97.6	74	86.1
15. Encourage Patricia to self-exclude from gambling venues	117	70.5	133	80.6	60	69.8
16. Encourage Patricia to learn about the strategies that gambling providers use to keep people gambling	104	63.8	129	79.1	67	77.9
18. Tell Patricia that there is effective professional help available for gambling problem	148	90.2	161	97.6	79	91.9
19. Encourage Patricia to seek professional help for her gambling	150	90.9	161	97.6	81	94.2
20. Suggest she leave bank cards and credit cards at home if she is going to a gambling venue	102	61.8	143	86.7	74	86.1
Mastery on intended recommended actions^b^	113	69.8	150	92.6	67	77.9

a. Participant rated they were “Likely” or “Very likely” to do action

b. Participants rated they were “Likely or “Very likely” to do at least 9 recommended actions

#### Non-recommended actions

Table 5 shows the proportion of participants who intended to avoid doing each of the non-recommended actions. Prior to the course, a little less than half of participants showed mastery for non-recommended actions. Concordance with actions that are not recommended improved after the course and at follow-up. These improvements were approximately medium in size (see Table 3). The most common action prior to the course was “telling the person what to do to change their gambling”, which showed limited improvement after training. The proportion of participants meeting mastery on non-recommended actions significantly improved from pre-course to post-course (*p* < 0.001), and pre-course to follow-up (*p* = 0.012).

**Table 5:** Number and percent of participants who intended to do actions that are not recommended to support Patricia^a^

Non-recommended actions	Pre		Post		Follow-up	
	n	%	n	%	N	%
2. Wait and see if her problems go away	11	6.7	2	1.2	1	1.2
3. Wait and see if her problems get worse	12	7.4	6	3.7	2	2.3
4. Wait and see if Patricia says that she thinks she might have gambling problem	25	15.6	14	8.7	6	7.0
7. Tell Patricia what to do to change her gambling	37	22.6	31	19.3	16	18.6
9. Tell Patricia she should stop gambling	28	17.1	8	4.9	7	8.1
10. Go gambling with Patricia to show her how to gamble responsibly	6	3.7	2	1.2	2	2.3
11. Tell her you won’t meet with her again until she stops gambling	3	1.8	2	1.2	1	1.2
17. Agree to give Patricia a loan if she promises to cut down or stop her gambling	6	3.7	3	1.9	2	2.3
Mastery on intended non-recommended actions^b^	75	47.2	115	72.8	55	64.0

a. Participant rated they were “Likely” or “Very likely” to do action

b. Participants rated they were “Unlikely” or “Very unlikely” to do at least 7 non-recommended actions

### Confidence in intended helping behaviours

Participants were asked about their confidence to assist Patricia. Mean responses increased from 2.71 (SD 0.96) before the course to 3.95 (SD 0.67) afterward and were 3.86 (SD 0.79) at follow-up. These increases in confidence were large in size and statistically significant (*p* < 0.001; see Table 3).

### Confidence in and quality of actual helping behaviours

Before the training, 108 participants (62.6%) reported knowing a person showing signs of problem gambling, including 38 who knew two or three people, and 25 who knew four or more people. Of the 87 participants who provided data at follow-up, 58 participants (66.7%) reported knowing someone with problem gambling, including 20 who knew two or three people and 19 who knew four or more people. There were 54 participants who reported knowing a person with problem gambling at both timepoints. These individuals were most commonly a family member (20.4% pre-course, 22.4% at follow-up) or friend (24.1% pre-course, 20.7% at follow-up) of the participant.

Of those participants who knew a person with problem gambling in the 6 months prior to the course, a small minority did not take any action to support the person (5%). At follow-up, all participants who had known a person with problem gambling since completing the course took at least one action to assist the person.

**Fig. 1:**
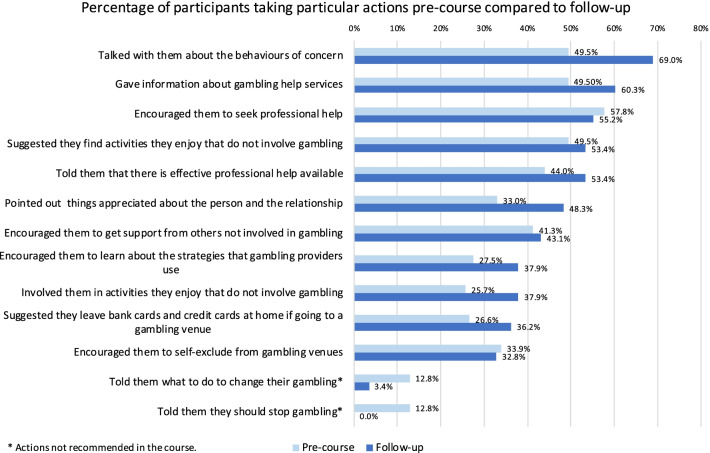
Percentage of participants taking particular actions at pre-course compared to follow-up

Figure 1 shows the percentage of participants taking particular actions before the course and at follow-up. As shown in Table 6, no single recommended action was done by more than 70% of participants. The most common actions reported pre-course were encouraging the person to get professional help (57.8%), giving them information about gambling services (49.5%), talking with them about their behaviours of concern (49.5%) and suggesting they find activities they enjoy that do not involve gambling (49.5%). These actions were also the most common actions at follow-up, with an additional common action at follow-up of telling the person that effective professional help is available (53.4%). The largest increase (of 20%) from pre-course to follow-up was talking with the person about behaviours of concern.

**Table 6:** Number and percent of participants who took actions to support a person that are recommended by MHFA training

Recommended actions	Pre (n = 109)		Follow-up (n = 58)	
	n	%	n	%
5. I gave them some information about gambling help services	54	49.5	35	60.3
6. I pointed out some things that I appreciate about the person and my relationship with them	36	33.0	28	48.3
8. I talked with them about the behaviours that were concerning me	54	49.5	40	69.0
12. I suggested they find activities they enjoy that do not involve gambling	54	49.5	31	53.4
13. I involved them in activities they enjoy that do not involve gambling	28	25.7	22	37.9
14. I encouraged them to get support from other people (e.g., family or friends) who are not involved in gambling	45	41.3	25	43.1
15. I encouraged the person to self-exclude from gambling venues	37	33.9	19	32.8
16. I encouraged them to learn about the strategies that gambling providers use to keep people gambling	30	27.5	22	37.9
17. I suggested they leave bank cards and credit cards at home if they are going to a gambling venue	29	26.6	21	36.2
19. I told them that there is effective professional help available for gambling problems	48	44.0	31	53.4
20. I encouraged them to seek professional help for their gambling	63	57.8	32	55.2
Actions concordant with guidelines/training^a^	11	10.1	10	17.2

a. Participants reported they had done at least 9 recommended actions

Prior to the course, a small minority of participants showed mastery of recommended actions. From pre-course to follow-up there was a small increase in help provided that was concordant with recommended actions, but this was not significant (see Table 3).

Most participants showed mastery on non-recommended actions prior to the course (indicating most did none of the non-recommended actions, see Table 7). The most common actions done pre-course were telling the person they should stop gambling (12.8%) and telling them what to do to change their gambling (12.8%; see Fig. 1). Very few participants reported doing any of the non-recommended actions at follow-up. From pre-course to follow-up there was a significant increase in concordant responses for non-recommended actions, which was a medium effect (see Table 3).

**Table 7:** Number and percent of participants who took actions to support a person that are not recommended by MHFA training

Non-recommended actions	Pre (n = 109)		Follow-up (n = 58)	
	n	%	n	%
2. I waited to see if their problems went away	3	2.8	0	0.0
3. I waited to see if their problems got worse	3	2.8	0	0.0
4. I waited to see if the person said that they think they might have gambling problems	10	9.2	2	3.4
7. I told them what to do to change their gambling	14	12.8	2	3.4
9. I told them they should stop gambling	14	12.8	0	0.0
10. I went gambling with the person to show them how to gamble responsibly	1	0.9	1	1.7
11. I told them I would not have contact with them until they stopped gambling	0	0.0	0	0.0
18. I agreed to give the person a loan if they promised to cut down or stop their gambling	2	1.8	0	0.0
21. I did not do anything	6	5.5	0	0.0
Actions concordant with guidelines^a^	97	89.0	57	98.3

a. Participants reported they had avoided doing at least 8 actions that are not recommended

The proportion of participants meeting mastery on recommended actions did not significantly improve from pre-course to follow-up (*p* = 0.185), but there was a significant increase in the proportion of participants meeting mastery for non-recommended actions (*p* = 0.033).

Participants also rated how confident they were in their ability to help the person with problem gambling. Confidence improved significantly from pre-course to follow-up and this effect was large in size (see Table 3).

### Course satisfaction

Table 8 shows that participants rated all aspects of the course quite highly, with average scores of at least 4 out of 5 on most course satisfaction questions. The exception to this was a question asking how new the course content was; the average score was 3.7. This is consistent with the rates of previous experience and training reported by participants. It is also in line with evaluations of other MHFA courses, and is unlikely to reflect a poor rating of the course content. With an average rating of 3.9 out of 4, participants were also highly likely to recommend the course to others.

**Table 8:** Mean scores on course satisfaction measures

Course satisfaction measures	Range	n	Mean (SD)
How new was the information in the course to you?	1 (not at all new) to 5 (mostly new)	165	3.7 (0.91)
How much of the information in the program did you understand?	1 (none of it) to 5 (most of it)	165	4.9 (0.39)
How well did the instructor present the program?	1 (very poorly) to 5 (very well)	165	4.9 (0.31)
How relevant was the content for you?	1 (not very much) to 5 (very much)	165	4.5 (0.76)
Please rate how much you liked the following parts of the program:			
The handbook	1 (not very much) to 5 (very much)	165	4.6 (0.78)
The PowerPoint slides	1 (not very much) to 5 (very much)	165	4.6 (0.70)
The films	1 (not very much) to 5 (very much)	165	4.7 (0.53)
The activities	1 (not very much) to 5 (very much)	165	4.4 (0.79)
Would you recommend the course to others?	1 (definitely not) to 4 (definitely)	165	3.9 (0.33)

**Fig. 2:**
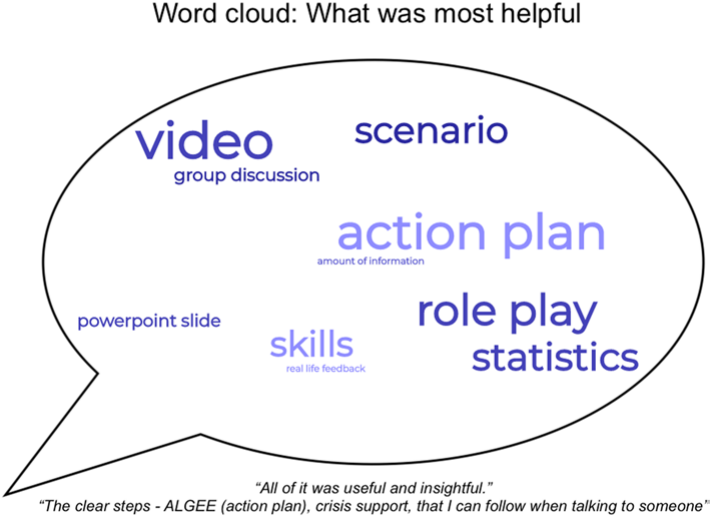
Summary of qualitative data on what was helpful

**Fig. 3:**
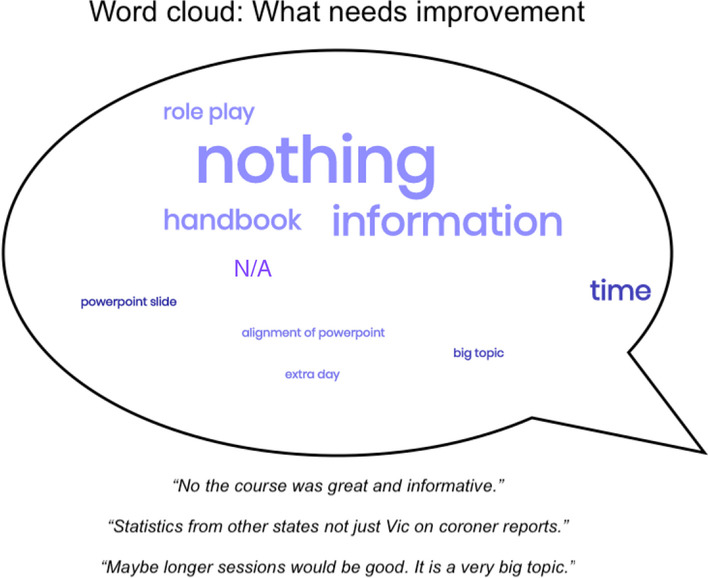
Summary of qualitative data on what could be improved

Qualitative data (see Figs. 2 and 3 for a summary) suggests that participants found the skills learnt in the course, the films shown, and the number of explanations and information provided to be the most helpful aspects of the course. Interactive components such as role plays and group discussions were also perceived as helpful. Most participants who responded to the question of what aspects of the course could be improved said that no improvements were needed. A small minority suggested the inclusion of more information (e.g. national statistics, details of other mental health problems), and that the course should be longer.

## Discussion

This evaluation of the *Conversations About Gambling* course used a pre-course, post-course and 6-month follow-up design to measure the effects of the course on participants’ knowledge about gambling and gambling problems, desire for social distance, confidence in supporting someone with problem gambling, and use of the skills taught in the course. It also explored participant satisfaction with the course. This study found that the course increased knowledge and confidence, decreased stigma and improved the quality of intentions to help someone experiencing gambling problems. These results are similar to the findings from other evaluations of MHFA courses [18, 19, 27, 28]. Participants were also highly satisfied with all elements of the course.

The evidence that the *Conversations About Gambling* course positively influences actual helping behaviour is less clear. This was also the case in a recent evaluation of the Mental Health First Aid for the Suicidal Person course, and like that study, this could be partially due to a low response rate, leading to insufficient statistical power to assess this item at follow-up [28]. It is also possible that the *Conversations About Gambling* course does not lead to substantial changes in participants’ behaviour. Future research could aim to determine which explanation is most likely to account for these results. This could be achieved by basing power calculations on numbers needed to assess changes in behavioural outcomes, and employing study designs that can capture more instances of first aid provision, e.g., ecological momentary assessment methods.

Prior to the course, participants showed reasonable knowledge about gambling and gambling problems, and improvements were still significant both immediately after the course and six months later. Participants also had a reasonably high quality of helping intentions before the course, which improved post-course. However, unlike the sustained improvements in participants' knowledge, these improvements in helping intentions were not sustained at follow-up. Future reviews of the course curriculum could address this, perhaps by increasing the amount of practise each participant is involved in during the course, e.g. through additional role-plays or scenario-based learning. Refresher courses designed to maintain knowledge and skills should also be considered. Four-hour refresher courses for Youth, Standard (adult) and Aboriginal and Torres Strait Islander MHFA are currently offered every 3 years. The *Conversations About Gambling* course may also benefit from the development of short booster courses that can be delivered within months after the main course.

The most common non-recommended action prior to the course was “Tell Patricia what to do to change her gambling,” and this action showed limited improvement after training. Given the wide-ranging negative consequences one might witness in relation to problem gambling [4], this response to someone is understandable. However, telling the person what to do to change their gambling is contrary to the best available evidence. The *Conversations About Gambling* course recommends making suggestions on what the person might do using a non-judgemental approach, rather than telling them what to do [20]. Under a reactance theory framework [29], the way this discussion on changing gambling behaviour is broached is important because resistance to change may be activated if the person perceives that a freedom is being threatened. This may then reduce motivation for the person to change their gambling or seek necessary treatment. It should be noted that although telling the person what to do to change their gambling was the most common non-recommended action, and the one that showed least improvement, less than a quarter of participants expressed an intention to carry out this action. Additionally, and perhaps more importantly, very few participants reported employing this action with someone they knew at follow-up. Nevertheless, given the serious consequences of problem gambling and its strong link with mental health problems [4, 5], delays in receiving professional treatment should be avoided. It is therefore important to ensure that the *Conversations About Gambling* course is as effective as possible in improving all participants' understanding of, and helping intentions regarding, how best to discuss a person’s problem gambling with them. Future iterations of the course could explicitly address how to frame such conversations or options for changing behaviour, incorporating examples of different terms and how people might react to them (e.g., comparing “tell” with “suggest,” or “should” with “could”).

Helping someone they knew who showed signs of problem gambling was a common experience for course participants prior to the course and at follow-up. The types of assistance given at both time points were similar, especially for actions recommended by the course. However, after attending the course, very few participants reported doing any non-recommended actions. There was also a notable increase in talking about behaviours of concern amongst participants. Other actions that were more commonly taken by participants post-course included discussing with the person things that they appreciated about their relationship, involving them in activities that did not include gambling, providing information on gambling help services and encouraging the person to learn about strategies that gambling providers used. It appears that following the course participants were more confident in proactively talking with the person about their behaviour and offering support, and perhaps in some instances this was in place of telling the person outright to stop gambling and how they should change their behaviours (actions not recommended in the course that were used less by participants between the course and follow-up).

Actions that saw virtually no change from pre-course to follow-up included encouraging the person to seek professional help or support from others not involved in gambling and encouraging them to self-exclude from gambling venues. These recommended actions may be seen as unnecessary or inappropriate in some instances or not always the first port of call where other actions suffice (e.g. self-exclusion from a venue is not applicable if the person is gambling online). It is also possible that despite the teachings of the course, known help-seeking barriers are impacting the provision of help here, for example shame, embarrassment or stigma [13, 30]. However, given that the encouragement of professional help from family and friends is one of the strongest motivators for seeking treatment [11], a greater emphasis on these areas may be needed in future updates to the *Conversations About Gambling* course. To inform these updates, future research could investigate the reasons why course participants who helped a person experiencing gambling problems chose, or avoided taking, specific actions during their interaction.

The finding that improvements in mastery of helping intentions post-course were not wholly sustained at follow-up, and improvements in mastery of actual actions taken were limited, suggests that future updates to the *Conversations About Gambling* course should provide more opportunities for course participants to practise their skills to maintain mastery over time. A randomised controlled trial would also provide a clearer picture on whether the course effectively promotes helpful and recommended actions.

This research responds to calls in the literature for community training about gambling and problem gambling [11, 15], with the findings suggesting that such training benefits community members by increasing knowledge, reducing stigmatising attitudes, and improving some aspects of support offered to a person with problem gambling. Courses such as *Conversations About Gambling* may be one way of increasing awareness of the social cost of gambling [31], its widespread effects on people other than the gambler [4, 32], and the availability of effective treatments for problem gambling and comorbid mental health problems [33]. Future research could explore the social and economic impacts of implementing and scaling up community training programs, evaluating whether they can increase rates of help-seeking for people with problem gambling or reduce rates of harms such as financial hardship, intimate partner and family violence, and legal difficulties.

### Limitations

This research is not without limitations. There was no control group, so changes may be attributable to factors that were not assessed in this evaluation. It is not known whether gambling behaviour was changed in the person who received help. A low response rate at follow-up made it difficult to draw clear conclusions about whether the course improved actual first aid actions taken to support someone experiencing problem gambling. The sample was primarily female, reasonably well educated, did not typically speak a language other than English at home and attended the course in a professional capacity, so it is unclear how well these results would generalise to participant groups with different demographic profiles. Finally, it is unclear whether implementing and evaluating the *Conversations About Gambling* course in a different country would produce similar findings. Although the course curriculum was developed for the Australian context, the original Delphi expert consensus study involved panellists from several high income countries whose populations may also benefit from this course. Although the evaluation findings of longer, more general MHFA courses are similar across countries [20], future research should assess whether this holds for specialised shorter courses, and therefore whether the results of this research are generalisable.

## Conclusions

The release of MHFA Australia’s *Conversations About Gambling* course responds to calls in the literature for educational courses that teach members of the public how to recognise and respond to signs of problem gambling in a person close to them [11, 15]. The results of this study provide initial evidence that the *Conversations About Gambling* course is effective at reducing stigma towards people experiencing problem gambling, increasing participants’ knowledge and confidence, and improving the quality of some first aid actions in line with expert recommendations. Further research is needed to better understand: (a) how long the course’s effects on knowledge, stigmatising attitudes and confidence persist for; (b) how to further improve participants’ first aid actions towards a person experiencing problem gambling; (c) whether the effects of the course on participants’ behaviour can be sustained over time; (d) the effects of participants’ first aid on the behaviour of the person experiencing problem gambling; and (e) whether the *Conversations About Gambling* course demonstrates similar results in groups with different demographic characteristics.

## Supplementary Information

**Additional file 1**. Merged surveys: T1T2T3surveys.pdf

## Data Availability

The datasets used and analysed during the current study are available from the corresponding author on reasonable request.

## Abbreviation

MHFA: Mental Health First Aid

## References

[1] NSW Office of Responsible Gambling. About responsible gambling. 2020 [cited 2020 Dec 10]. Available from: https://www.responsiblegambling.nsw.gov.au/about-gambling/about-responsible-gambling

[2] Neal P, Delfabbro P, O’Neil M. Problem gambling and harm: towards a national definition. Melbourne: Office of Gaming and Racing, Victorian Government Department of Justice. 2005.

[3] Dowling NA, Youssef GJ, Jackson AC, Pennay DW, Francis KL, Pennay A (2016). National estimates of Australian gambling prevalence: Findings from a dual-frame omnibus survey. Addiction.

[4] Browne M, Langham E, Rawat V, Greer N, Li E, Rose J, et al. Assessing gambling-related harm in Victoria. Victorian Responsible Gambling Foundation. 2016.

[5] Lorains FK, Cowlishaw S, Thomas SA. Prevalence of comorbid disorders in problem and pathological gambling: systematic review and meta-analysis of population surveys. 2010;106:490–8. 10.1111/j.1360-0443.2010.03300.x 21210880

[6] Black DW, Coryell W, Crowe R, McCormick B, Shaw M, Allen J (2015). Suicide ideations, suicide attempts, and completed suicide in persons with pathological gambling and their first-degree relatives. Suicide Life-Threatening Behav.

[7] Patford J (2009). For worse, for poorer and in ill health: How women experience, understand and respond to a partner’s gambling problems. Int J Ment Health Addict.

[8] Thomas A, Delfabbro P, Armstrong AR. Validation study of in-venue problem gambler indicators. Gambling Research Australia; 2014. Available from: https://www.gamblingresearch.org.au/sites/default/files/2019-10/Validationstudyofin-venueproblemgamblerindicators2014.pdf

[9] Delfabbro P, Thomas A, Armstrong A (2016). Observable indicators and behaviors for the identification of problem gamblers in venue environments. J Behav Addict.

[10] Delfabbro P, Thomas A, Armstrong A (2018). Gender differences in the presentation of observable risk indicators of problem gambling. J Gambl Stud.

[11] Hing N, Nuske E, Gainsbury S. Gamblers at-risk and their help-seeking behaviour. Melbourne: Gambling Research Australia; 2012.

[12] Suurvali H, Cordingley J, Hodgins DC, Cunningham J (2009). Barriers to seeking help for gambling problems: a review of the empirical literature. J Gambl Stud.

[13] Hing N, Russell AMT. How anticipated and experienced stigma can contribute to self-stigma: The case of problem gambling. Front Psychol. 2017;8(235). 10.3389/fpsyg.2017.00235 PMC5318456 28270787

[14] Slutske WS. Natural recovery and treatment-seeking in pathological gambling: Results of two U.S. national surveys. Am J Psychiatry. 2006;163(2):297–302. 10.1176/appi.ajp.163.2.297 16449485

[15] Evans L, Delfabbro PH (2005). Motivators for change and barriers to help-seeking in Australian problem gamblers. J Gambl Stud.

[16] Hare S. A study of gambling in Victoria: Problem gambling from a public health perspective. Victorian Responsible Gambling Foundation. Melbourne: Department of Justice, Victoria; 2009. Available from: http://scholar.google.com/scholar?hl=en&btnG=Search&q=intitle:A+STUDY+OF+GAMBLING+IN+VICTORIA+PROBLEM+GAMBLING+FROM+A+PUBLIC+HEALTH#1

[17] Jorm AF, Ross AM. Guidelines for the public on how to provide mental health first aid: narrative review. BJPsych Open. 2018;4(6). 10.1192/bjo.2018.58 PMC6235998 30450221

[18] Hadlaczky G, Hökby S, Mkrtchian A, Carli V, Wasserman D. Mental health first aid is an effective public health intervention for improving knowledge, attitudes, and behaviour: a meta-analysis. Int Rev Psychiatry. 2014;26(4). 10.3109/09540261.2014.924910 25137113

[19] Morgan AJ, Ross A, Reavley NJ. Systematic review and meta-analysis of mental health first aid training: effects on knowledge, stigma, and helping behaviour. PLoS One. 2018;13(5). 10.1371/journal.pone.0197102 PMC5979014 29851974

[20] Bond KS, Jorm AF, Miller HE, Rodda SN, Reavley NJ, Kelly CM, et al. How a concerned family member, friend or member of the public can help someone with gambling problems: a Delphi consensus study. BMC Psychol. 2016;4(1). 10.1186/s40359-016-0110-y PMC4739356 26842544

[21] Mental Health First Aid Australia. Gambling Problems: MHFA Guidelines. Melbourne; 2015.

[22] Kelly L. Conversations about Gambling: Course Handbook. Melbourne: Mental Health First Aid; 2018.

[23] Link BG, Phelan JC, Bresnahan M, Stueve A, Pescosolido BA (1999). Public conceptions of mental illness: labels, causes, dangerousness, and social distance. Am J Public Health.

[24] Subkoviak MJ (1988). A practitioner’s guide to computation and interpretation of reliability indices for mastery tests. J Educ Meas.

[25] Salim A, Mackinnon A, Christensen H, Griffiths K (2008). Comparison of data analysis strategies for intent-to-treat analysis in pre-test-post-test designs with substantial dropout rates. Psychiatry Res.

[26] Cohen J. A power primer. Psychol Bull. 1992;112(1). 10.1037//0033-2909.112.1.155 19565683

[27] Armstrong G, Sutherland G, Pross E, Mackinnon A, Reavley N, Jorm AF. Talking about suicide: An uncontrolled trial of the effects of an Aboriginal and Torres Strait Islander mental health first aid program on knowledge, attitudes and intended and actual assisting actions. PLoS One. 2020;15(12):e0244091. 10.1371/journal.pone.0244091 PMC7746176 33332464

[28] Bond KS, Cottrill FA, Mackinnon A, Morgan AJ, Kelly CM, Armstrong G (2021). Effects of the Mental Health First Aid for the Suicidal Person course on beliefs about suicide, stigmatising attitudes, confidence to help, and intended and actual helping actions: an evaluation. Int J Ment Health Syst.

[29] Brehm SS, Brehm JW, Brehm SS, Brehm JW (1981). Reactance theory and control. Psychological reactance: a theory of freedom and control.

[30] Gainsbury S, Hing N, Suhonen N. Professional help-seeking for gambling problems: Awareness, barriers and motivators for treatment. J Gambl Stud. 2014;30(2). 10.1007/s10899-013-9373-x 23494244

[31] Productivity Commission. Gambling. Report no. 50. Canberra; 2010. Available from: https://www.pc.gov.au/inquiries/completed/gambling-2010/report

[32] Goodwin BC, Browne M, Rockloff M, Rose J (2017). A typical problem gambler affects six others. Int Gambl Stud.

[33] Quilty LC, Wardell JD, Thiruchselvam T, Keough MT, Hendershot CS. Brief interventions for problem gambling: a meta-analysis. PLoS One. 2019;14(4):e0214502. 10.1371/journal.pone.0214502 PMC6469774 30995229

