# Supplementary material for: Evaluation of the Conversations About Gambling Mental Health First Aid course: effects on knowledge, stigmatising attitudes, confidence and helping behaviour
Source: BMC Psychol. 2022 Mar 24;10:78. doi: 10.1186/s40359-022-00785-w (PMC8943993; doi:10.1186/s40359-022-00785-w)
Supplement: Supplementary file 1 — Additional file 1. Merged surveys: T1T2T3surveys.pdf [file 40359_2022_785_MOESM1_ESM.pdf]

Evaluating the Mental Health First Aid course for Gambling Problems

(T1)

Information about you

Please provide us with your name, phone number and email address. (We ask for this information so that we can match your three surveys and send you the final survey. Once your surveys are matched, your name is deleted from the information in the survey. Only the research officers have access to identifying information and ***we will not share your information with anyone***.)

1. Name

2. Email *(If you would prefer we send you a paper copy of the 6-month follow-up survey please give us your postal address.)*

3. Phone number

4. What is your gender?

☐ Male

☐ Female

☐ I prefer to self-describe (please state)

5. What was your age at your last birthday? (You must be 18 or older to participate in this research.)

6. What is the highest level of education you have completed?

☐ Year 9 or lower

☐ Year 10, 11, or 12

☐ Certificate, Trade or Apprenticeship

☐ University

7. Are you:

☐ Aboriginal

☐ Torres Strait Islander

☐ Both

☐ Neither

8. What is the post code of where you live?

9. Do you speak a language other than English as your first language at home?

☐ No

☐ Yes

10. Have you done any previous training about gambling problems?

☐ No

☐ Yes, please let us know what training you have done:

11. Have you done any previous mental health training (including short courses or professional training)? If yes, what have you done:

12. Do you have any personal or professional experience with gambling problems? *Please tick all that apply.*

☐ Gambling problems in clients or customers

☐ Gambling problems in a colleague

☐ Gambling problems in myself

☐ Gambling problems in my friends

☐ Gambling problems in my family

☐ Gambling problems in my broader community

☐ None of the above

☐ I'd rather not say

13. Why are you interested in learning mental health first aid for gambling problems? *Please tick all that apply.*

☐ As part of continuing education for my workplace/profession

☐ As part of training for a volunteer job

☐ I currently support/know someone with gambling problems

☐ In the past, I have had contact with someone with gambling problems

☐ I have had gambling problems

☐ Other, please specify:

Patricia (*Imagine Patricia is someone you know and care about.*)

The following section concerns a hypothetical person called Patricia. The description below outlines how they have been recently.

You and Patricia have been friends for years and get together every two weeks for coffee or a meal. In the last few months she has not seemed like her usual self. She seems a bit flat and is much less talkative than usual. You know that Patricia recently changed jobs due to some 'personal' problems with the previous employer. You have heard she was going to the casino on her lunch break and getting back to work late.

(Continued on next page)

You also know that Patricia and her partner have been having more frequent disagreements about money and time she spends at the casino. Over the last three months Patricia has missed a few of your regular get-togethers and when you do meet up she seems preoccupied by betting apps on her phone or talks about time spent at the pokies more than anything else. Patricia has recently asked you for a loan to help her get to pay day.

14. How confident are you in your ability to help Patricia? (Circle the number that represents your answer.)

1	2	3	4	5
Not at all confident	A little bit confident	Moderately confident	Quite a bit confident	Extremely confident

15. How likely would you be to do the following to help Patricia:

	**Very unlikely**	**Unlikely**	**Neither unlikely or likely**	**Likely**	**Very likely**
Ask someone else to talk to her.					
Wait and see if her problems go away.					
Wait and see if her problems get worse.					
Wait and see if Patricia says that she thinks she might have gambling problems.					
Give Patricia some information about gambling help services.					
Point out some things that you appreciate about Patricia and your relationship with her.					
Tell Patricia what to do to change her gambling.					
Talk with Patricia about the behaviours that are concerning you.					
Tell Patricia she should stop gambling.					
Go gambling with Patricia to show her how to gamble responsibly.					

15. How likely would you be to do the following to help Patricia (cont.):

	Very unlikely	Unlikely	Neither unlikely or likely	Likely	Very likely
Tell her you won't meet with her again until she stops gambling.					
Suggest she find activities she enjoys that do not involve gambling.					
Involve Patricia in activities that she enjoys that do not involve gambling.					
Encourage Patricia to get support from other people (e.g. family or friends) who are not involved in gambling.					
Encourage Patricia to self-exclude from gambling venues.					
Encourage Patricia to learn about the strategies that gambling providers use to keep people gambling.					
Agree to give Patricia a loan if she promises to cut down or stop her gambling.					
Tell Patricia that there is effective professional help available for gambling problems.					
Encourage Patricia to seek professional help for her gambling.					
Suggest she leave bank cards and credit cards at home if she is going to a gambling venue.					

16. The following questions ask how you would feel about spending time with *__a person with a problem like__* Patricia's. Would you be happy to...

	Definitely not	Probably not	Not sure	Yes, probably	Yes, definitely
Move next door to them?					
Spend an evening socialising with them?					
Make friends with them?					
Work closely with them on a project?					
Have them marry into your family?					
Employ them?					
Vote for a politician if you knew they had suffered a problem like Patricia's?					

Information about gambling and gambling problems

17. The next section contains statements about gambling problems. Please indicate whether you agree or disagree with each statement, or if you don't know.

Most people who gamble do not have a problem with gambling.	DISAGREE	AGREE	DON'T KNOW
When talking to someone about their gambling problems it is best to let them know clearly that you disapprove of gambling.	DISAGREE	AGREE	DON'T KNOW
People with gambling problems are likely to experience common mental health problems, such as depression, anxiety and substance abuse problems.	DISAGREE	AGREE	DON'T KNOW
Gambling problems are often motivated by the desire to escape problems or unpleasant emotions.	DISAGREE	AGREE	DON'T KNOW
People who experience gambling problems are at increased risk of suicide.	DISAGREE	AGREE	DON'T KNOW

17. The next section contains statements about gambling problems (cont.).
Please indicate whether you agree or disagree with each statement, or if you don't know.

If a person with gambling problems is suicidal, your initial focus should be on helping them with financial problems first.	DISAGREE	AGREE	DON'T KNOW
It can be helpful to use shame or guilt to encourage a person to stop problem gambling.	DISAGREE	AGREE	DON'T KNOW
Many people with gambling problems will experience serious financial hardship before they choose to seek help.	DISAGREE	AGREE	DON'T KNOW
A person may have gambling problems even if their gambling has not led to financial problems.	DISAGREE	AGREE	DON'T KNOW
A person with gambling problems may not look after their health, e.g. may not take their medication.	DISAGREE	AGREE	DON'T KNOW
Most people with gambling problems access professional help.	DISAGREE	AGREE	DON'T KNOW
The goal of treatment of gambling problems should always be abstinence from all gambling.	DISAGREE	AGREE	DON'T KNOW
Most people require professional help to recover from gambling problems.	DISAGREE	AGREE	DON'T KNOW
People with gambling problems need to experience a crisis before they can benefit from seeking help.	DISAGREE	AGREE	DON'T KNOW
A person with gambling problems may not want to stop gambling because it may create a gap in their life.	DISAGREE	AGREE	DON'T KNOW
People with gambling problems who try to stop on their own are more likely to relapse.	DISAGREE	AGREE	DON'T KNOW
If a person with gambling problems relapses, it proves they cannot recover from their problems.	DISAGREE	AGREE	DON'T KNOW
To show the person that you are on their side, you should consider offering them a loan to cover debts.	DISAGREE	AGREE	DON'T KNOW

17. The next section contains statements about gambling problems (cont.). Please indicate whether you agree or disagree with each statement, or if you don't know.

If the person reacts negatively when you raise your concerns about their gambling problems, you should persist in talking to them about their gambling until they accept they have a problem.	DISAGREE	AGREE	DON'T KNOW
'Interventions', where a group of people confront the person about their gambling problems, can be very helpful in getting the person to change.	DISAGREE	AGREE	DON'T KNOW

People you have been concerned about

18. In the past 6 months, how many people have you had contact with who you were concerned may have gambling problems?

☐ None ***(you do not need to complete any more of the survey)***

☐ One person ***(please continue with the next question)***

☐ Two or three people ***(please continue with the next question)***

☐ Four or more people ***(please continue with the next question)***

If you had contact with more than one person, think about the person you had the most contact with.

19. What is the age of the person?

☐ under 18

☐ 19-29

☐ 30-39

☐ 40-49

☐ 50-59

☐ 60-69

☐ 70-79

☐ 80 or over

20. What was their gender:

☐ Male

☐ Female

☐ Other

☐ Unsure

21.What was their relationship to you?

☐ Family member

☐ Friend

☐ Work colleague

☐ Acquaintance

☐ Stranger

☐ Client/patient

☐ Other, please specify:

22. Did you do any of the following?

☐ I asked someone else to talk to them.

☐ I waited to see if their problems went away.

☐ I waited to see if their problems got worse.

☐ I waited to see if the person said that they think they might have gambling problems.

☐ I gave them some information about gambling help services.

☐ I pointed out some things that I appreciate about the person and my relationship with them.

☐ I told them what to do to change their gambling.

☐ I talked with them about the behaviours that were concerning me.

☐ I told them they should stop gambling.

☐ I went gambling with the person to show them how to gamble responsibly.

☐ I told them I would not have contact with them until they stopped gambling.

☐ I suggested they find activities they enjoy that do not involve gambling.

☐ I involved them in activities they enjoy that do not involve gambling.

☐ I encouraged them to get support from other people (e.g. family or friends) who are not involved in gambling.

☐ I encouraged the person to self-exclude from gambling venues.

☐ I encouraged them to learn about the strategies that gambling providers use to keep people gambling.

22. Did you do any of the following (cont.)?

☐ I suggested they leave bank cards and credit cards at home if they are going to a gambling venue.

☐ I agreed to give the person a loan if they promised to cut down or stop their gambling.

☐ I told them that there is effective professional help available for gambling problems.

☐ I encouraged them to seek professional help for their gambling.

☐ I did not do anything ***(please go to question 24 on this page).***

☐ I did something else. Please let us know what you did:

23. How confident were you in your ability to help the person?

1	2	3	4	5
Not at all confident	A little bit confident	Moderately confident	Quite a bit confident	Extremely confident

24. *If you didn't try to help the person*, are there any particular reasons that you did not try to help the person with this problem? If so, please describe these reasons.

Thank you for completing this survey!

Evaluating the Mental Health First Aid course for Gambling Problems

(T2)

Information about you

Please provide us with your name, phone number and email address. (We ask for this information so that we can match your three surveys and send you the final survey. Once your surveys are matched, your name is deleted from the information in the survey. Only the research officers have access to identifying information and ***we will not share your information with anyone***.)

1. Name

2. Email

3. Phone number

Patricia *(Imagine Patricia is someone you know and care about.)*

The following section concerns a hypothetical person called Patricia. The description below outlines how they have been recently.

You and Patricia have been friends for years and get together every two weeks for coffee or a meal. In the last few months she has not seemed like her usual self. She seems a bit flat and is much less talkative than usual. You know that Patricia recently changed jobs due to some 'personal' problems with the previous employer. You have heard she was going to the casino on her lunch break and getting back to work late.

You also know that Patricia and her partner have been having more frequent disagreements about money and time she spends at the casino. Over the last three months Patricia has missed a few of your regular get-togethers and when you do meet up she seems preoccupied by betting apps on her phone or talks about time spent at the pokies more than anything else. Patricia has recently asked you for a loan to help her get to pay day.

4. How confident are you in your ability to help Patricia? (Circle the number that represents your answer.)

1<br>Not at all confident	2<br>A little bit confident	3<br>Moderately confident	4<br>Quite a bit confident	5<br>Extremely confident

5. How likely would you be to do the following to help Patricia:

	**Very unlikely**	**Unlikely**	**Neither unlikely or likely**	**Likely**	**Very likely**
Ask someone else to talk to her.					
Wait and see if her problems go away.					
Wait and see if her problems get worse.					
Wait and see if Patricia says that she thinks she might have gambling problems.					
Give Patricia some information about gambling help services.					
Point out some things that you appreciate about Patricia and your relationship with her.					
Tell Patricia what to do to change her gambling.					
Talk with Patricia about the behaviours that are concerning you.					
Tell Patricia she should stop gambling.					
Go gambling with Patricia to show her how to gamble responsibly.					

5. How likely would you be to do the following to help Patricia (cont.):

	Very unlikely	Unlikely	Neither unlikely or likely	Likely	Very likely
Tell her you won't meet with her again until she stops gambling.					
Suggest she find activities she enjoys that do not involve gambling.					
Involve Patricia in activities that she enjoys that do not involve gambling.					
Encourage Patricia to get support from other people (e.g. family or friends) who are not involved in gambling.					
Encourage Patricia to self-exclude from gambling venues.					
Encourage Patricia to learn about the strategies that gambling providers use to keep people gambling.					
Agree to give Patricia a loan if she promises to cut down or stop her gambling.					
Tell Patricia that there is effective professional help available for gambling problems.					
Encourage Patricia to seek professional help for her gambling.					

5. How likely would you be to do the following to help Patricia (cont.):

	**Very unlikely**	**Unlikely**	**Neither unlikely or likely**	**Likely**	**Very likely**
Suggest she leave bank cards and credit cards at home if she is going to a gambling venue.					

6. The following questions ask how you would feel about spending time with *__a person with a problem like__* Patricia's. Would you be happy to...

	**Definitely not**	**Probably not**	**Not sure**	**Yes, probably**	**Yes, definitely**
Move next door to them?					
Spend an evening socialising with them?					
Make friends with them?					
Work closely with them on a project?					
Have them marry into your family?					
Employ them?					
Vote for a politician if you knew they had suffered a problem like Patricia's?					

<u>Information about gambling and gambling problems</u>

7. The next section contains statements about gambling problems. Please indicate whether you agree or disagree with each statement, or if you don't know.

Most people who gamble do not have a problem with gambling.	DISAGREE	AGREE	DON'T KNOW
When talking to someone about their gambling problems it is best to let them know clearly that you disapprove of gambling.	DISAGREE	AGREE	DON'T KNOW

7. The next section contains statements about gambling problems (cont.).
Please indicate whether you agree or disagree with each statement, or if you don't know.

People with gambling problems are likely to experience common mental health problems, such as depression, anxiety and substance abuse problems.	DISAGREE	AGREE	DON'T KNOW
Gambling problems are often motivated by the desire to escape problems or unpleasant emotions.	DISAGREE	AGREE	DON'T KNOW
People who experience gambling problems are at increased risk of suicide.	DISAGREE	AGREE	DON'T KNOW
If a person with gambling problems is suicidal, your initial focus should be on helping them with financial problems first.	DISAGREE	AGREE	DON'T KNOW
It can be helpful to use shame or guilt to encourage a person to stop problem gambling.	DISAGREE	AGREE	DON'T KNOW
Many people with gambling problems will experience serious financial hardship before they choose to seek help.	DISAGREE	AGREE	DON'T KNOW
A person may have gambling problems even if their gambling has not led to financial problems.	DISAGREE	AGREE	DON'T KNOW
A person with gambling problems may not look after their health, e.g. may not take their medication.	DISAGREE	AGREE	DON'T KNOW
Most people with gambling problems access professional help.	DISAGREE	AGREE	DON'T KNOW
The goal of treatment of gambling problems should always be abstinence from all gambling.	DISAGREE	AGREE	DON'T KNOW
Most people require professional help to recover from gambling problems.	DISAGREE	AGREE	DON'T KNOW
People with gambling problems need to experience a crisis before they can benefit from seeking help.	DISAGREE	AGREE	DON'T KNOW
A person with gambling problems may not want to stop gambling because it may create a gap in their life.	DISAGREE	AGREE	DON'T KNOW
People with gambling problems who try to stop on their own are more likely to relapse.	DISAGREE	AGREE	DON'T KNOW

7. The next section contains statements about gambling problems (cont.). Please indicate whether you agree or disagree with each statement, or if you don't know.

If a person with gambling problems relapses, it proves they cannot recover from their problems.	DISAGREE	AGREE	DON'T KNOW
To show the person that you are on their side, you should consider offering them a loan to cover debts.	DISAGREE	AGREE	DON'T KNOW
If the person reacts negatively when you raise your concerns about their gambling problems, you should persist in talking to them about their gambling until they accept they have a problem.	DISAGREE	AGREE	DON'T KNOW
'Interventions', where a group of people confront the person about their gambling problems, can be very helpful in getting the person to change.	DISAGREE	AGREE	DON'T KNOW

Feedback about the course

8. How new was the information in the course to you?

1<br>Not at all new	2	3<br>Somewhat new	4	5<br>Mostly new

9. How much of the information in the program did you understand?

1<br>None of it	2	3<br>Some of it	4	5<br>Most of it

10. How well did the instructor present the program?

1<br>Very poorly	2	3<br>Somewhat well	4	5<br>Very well

11. How relevant was the content for you?

1<br>Not very much	2	3<br>Somewhat	4	5<br>Very much

12. Please rate how much you liked the following parts of the program:

	Not very much		Somewhat		Very much
**Handbook**	☐	☐	☐	☐	☐
**PowerPoint slides**	☐	☐	☐	☐	☐
**Films**	☐	☐	☐	☐	☐
**Activities**	☐	☐	☐	☐	☐

13. What aspects of the course did you find most helpful?

14. Is there anything in the course that could be improved?

15. Would you recommend the course to others?

1	2	3	4
Definitely not	Probably not	Probably	Yes, definitely

Please take this page with you.

Thank you for completing this survey!

If taking this survey brings up difficult emotions, you may wish to contact any of the following for free online or telephone counselling, available 24 hours a day, seven days a week.

Lifeline on 13 11 14

Suicide Call Back Service on 1300 659 467

Gambling Help Online on 1800 858 858 or www.gamblinghelponline.org.au

Evaluating the Mental Health First Aid course for Gambling Problems

(T3)

Information about you

Please provide us with your name and email address. (We ask for this information so that we can match your three surveys. Once your surveys are matched, your name is deleted from the information in the survey. Only the research officers have access to identifying information and ***we will not share your information with anyone***.)

1. Name

2. Email

Patricia (*Imagine Patricia is someone you know and care about.)*

The following section concerns a hypothetical person called Patricia. The description below outlines how they have been recently.

You and Patricia have been friends for years and get together every two weeks for coffee or a meal. In the last few months she has not seemed like her usual self. She seems a bit flat and is much less talkative than usual. You know that Patricia recently changed jobs due to some 'personal' problems with the previous employer. You have heard she was going to the casino on her lunch break and getting back to work late.

You also know that Patricia and her partner have been having more frequent disagreements about money and time she spends at the casino. Over the last three months Patricia has missed a few of your regular get-togethers and when you do meet up she seems preoccupied by betting apps on her phone or talks about time spent at the pokies more than anything else. Patricia has recently asked you for a loan to help her get to pay day.

3. How confident are you in your ability to help Patricia? (Circle the number that represents your answer.)

1<br>Not at all confident	2<br>A little bit confident	3<br>Moderately confident	4<br>Quite a bit confident	5<br>Extremely confident

4. How likely would you be to do the following to help Patricia:

	**Very unlikely**	**Unlikely**	**Neither unlikely or likely**	**Likely**	**Very likely**
Ask someone else to talk to her.					
Wait and see if her problems go away.					
Wait and see if her problems get worse.					
Wait and see if Patricia says that she thinks she might have gambling problems.					
Give Patricia some information about gambling help services.					
Point out some things that you appreciate about Patricia and your relationship with her.					
Tell Patricia what to do to change her gambling.					
Talk with Patricia about the behaviours that are concerning you.					
Tell Patricia she should stop gambling.					
Go gambling with Patricia to show her how to gamble responsibly.					

4. How likely would you be to do the following to help Patricia (cont.):

	Very unlikely	Unlikely	Neither unlikely or likely	Likely	Very likely
Tell her you won't meet with her again until she stops gambling.					
Suggest she find activities she enjoys that do not involve gambling.					
Involve Patricia in activities that she enjoys that do not involve gambling.					
Encourage Patricia to get support from other people (e.g. family or friends) who are not involved in gambling.					
Encourage Patricia to self-exclude from gambling venues.					
Encourage Patricia to learn about the strategies that gambling providers use to keep people gambling.					
Agree to give Patricia a loan if she promises to cut down or stop her gambling.					
Tell Patricia that there is effective professional help available for gambling problems.					
Encourage Patricia to seek professional help for her gambling.					
Suggest she leave bank cards and credit cards at home if she is going to a gambling venue.					

5. The following questions ask how you would feel about spending time with *__a person with a problem like__* Patricia's. Would you be happy to...

	**Definitely not**	**Probably not**	**Not sure**	**Yes, probably**	**Yes, definitely**
Move next door to them?					
Spend an evening socialising with them?					
Make friends with them?					
Work closely with them on a project?					
Have them marry into your family?					
Employ them?					
Vote for a politician if you knew they had suffered a problem like Patricia's?					

Information about gambling and gambling problems

6. The next section contains statements about gambling problems. Please indicate whether you agree or disagree with each statement, or if you don't know.

Most people who gamble do not have a problem with gambling.	DISAGREE	AGREE	DON'T KNOW
When talking to someone about their gambling problems it is best to let them know clearly that you disapprove of gambling.	DISAGREE	AGREE	DON'T KNOW
People with gambling problems are likely to experience common mental health problems, such as depression, anxiety and substance abuse problems.	DISAGREE	AGREE	DON'T KNOW
Gambling problems are often motivated by the desire to escape problems or unpleasant emotions.	DISAGREE	AGREE	DON'T KNOW
People who experience gambling problems are at increased risk of suicide.	DISAGREE	AGREE	DON'T KNOW

6. The next section contains statements about gambling problems (cont.).
Please indicate whether you agree or disagree with each statement, or if you don't know.

If a person with gambling problems is suicidal, your initial focus should be on helping them with financial problems first.	DISAGREE	AGREE	DON'T KNOW
It can be helpful to use shame or guilt to encourage a person to stop problem gambling.	DISAGREE	AGREE	DON'T KNOW
Many people with gambling problems will experience serious financial hardship before they choose to seek help.	DISAGREE	AGREE	DON'T KNOW
A person may have gambling problems even if their gambling has not led to financial problems.	DISAGREE	AGREE	DON'T KNOW
A person with gambling problems may not look after their health, e.g. may not take their medication.	DISAGREE	AGREE	DON'T KNOW
Most people with gambling problems access professional help.	DISAGREE	AGREE	DON'T KNOW
The goal of treatment of gambling problems should always be abstinence from all gambling.	DISAGREE	AGREE	DON'T KNOW
Most people require professional help to recover from gambling problems.	DISAGREE	AGREE	DON'T KNOW
People with gambling problems need to experience a crisis before they can benefit from seeking help.	DISAGREE	AGREE	DON'T KNOW
A person with gambling problems may not want to stop gambling because it may create a gap in their life.	DISAGREE	AGREE	DON'T KNOW
People with gambling problems who try to stop on their own are more likely to relapse.	DISAGREE	AGREE	DON'T KNOW
If a person with gambling problems relapses, it proves they cannot recover from their problems.	DISAGREE	AGREE	DON'T KNOW
To show the person that you are on their side, you should consider offering them a loan to cover debts.	DISAGREE	AGREE	DON'T KNOW

6. The next section contains statements about gambling problems (cont.). Please indicate whether you agree or disagree with each statement, or if you don't know.

If the person reacts negatively when you raise your concerns about their gambling problems, you should persist in talking to them about their gambling until they accept they have a problem.	DISAGREE	AGREE	DON'T KNOW
'Interventions', where a group of people confront the person about their gambling problems, can be very helpful in getting the person to change.	DISAGREE	AGREE	DON'T KNOW

People you have been concerned about

7. In the past 6 months, how many people have you had contact with who you were concerned may have gambling problems?

☐ None (***please go to question 14 on page 9)***

☐ One person (***please continue with the next question)***

☐ Two or three people (***please continue with the next question)***

☐ Four or more people (***please continue with the next question)***

If you had contact with more than one person, think about the person you had the most contact with.

8. What is the age of the person?

☐ under 18

☐ 19-29

☐ 30-39

☐ 40-49

☐ 50-59

☐ 60-69

☐ 70-79

☐ 80 or over

9. What was their gender:

☐ Male

☐ Female

☐ Other

☐ Unsure

10. What was their relationship to you?

☐ Family member

☐ Friend

☐ Work colleague

☐ Acquaintance

☐ Stranger

☐ Client/patient

☐ Other, please specify:

11. Did you do any of the following?

☐ I asked someone else to talk to them.

☐ I waited to see if their problems went away.

☐ I waited to see if their problems got worse.

☐ I waited to see if the person said that they think they might have gambling problems.

☐ I gave them some information about gambling help services.

☐ I pointed out some things that I appreciate about the person and my relationship with them.

☐ I told them what to do to change their gambling.

☐ I talked with them about the behaviours that were concerning me.

☐ I told them they should stop gambling.

☐ I went gambling with the person to show them how to gamble responsibly.

☐ I told them I would not have contact with them until they stopped gambling.

☐ I suggested they find activities they enjoy that do not involve gambling.

☐ I involved them in activities they enjoy that do not involve gambling.

☐ I encouraged them to get support from other people (e.g. family or friends) who are not involved in gambling.

☐ I encouraged the person to self-exclude from gambling venues.

11. Did you do any of the following? (cont.).

☐ I encouraged them to learn about the strategies that gambling providers use to keep people gambling.

☐ I suggested they leave bank cards and credit cards at home if they are going to a gambling venue.

☐ I agreed to give the person a loan if they promised to cut down or stop their gambling.

☐ I told them that there is effective professional help available for gambling problems.

☐ I encouraged them to seek professional help for their gambling.

☐ I did not do anything ***(please go to question 13 below).***

☐ I did something else, please let us know what you did:

12. How confident were you in your ability to help the person?

1	2	3	4	5
Not at all confident	A little bit confident	Moderately confident	Quite a bit confident	Extremely confident

Please go to question 14 on page 9.

13. <u>If you didn't try to help the person</u>, are there any particular reasons that you did not try to help the person with this problem? If so, please describe these reasons.

Feedback about the course

14. Have you recommended the course to others?

☐ No

☐ Yes

15. How much of the handbook have you read?

1<br>None of it	2<br>Part of it	3<br>Most of it	4<br>All of it

16. How easy was the handbook to understand?

1<br>Very difficult	2<br>Difficult	3<br>Neither difficult nor easy	4<br>Easy	5<br>Very easy

17. How much did you learn from the handbook?

1<br>Almost nothing	2<br>Not very much	3<br>A fair bit	4<br>A great deal

18. How useful was the handbook?

1<br>Not at all useful	2<br>Not very useful	3<br>Useful	4<br>Very useful

19. Do you think you will use the handbook in the future?

☐ No

☐ Yes

☐ Not sure

20. What have you done with the handbook? Please choose all that apply.

- ☐ Kept it
- ☐ Lent it to someone
- ☐ Given it away
- ☐ Thrown it away
- ☐ Lost it
- ☐ Don’t know

21. What did you like about the handbook?

22. What did you dislike about the handbook?

Please keep this page.

Thank you for completing this survey!

If taking this survey brings up difficult emotions, you may wish to contact any of the following for free online or telephone counselling, available 24 hours a day, seven days a week.

Lifeline on 13 11 14

Suicide Call Back Service on 1300 659 467

Gambling Help Online on 1800 858 858 or www.gamblinghelponline.org.au
